# Three inhibitory phenolic acids against common ragweed (*Ambrosia artemisiifolia* L.) had a minimal effect on maize growth *in vitro* and *in vivo*

**DOI:** 10.1371/journal.pone.0308825

**Published:** 2024-09-27

**Authors:** Laura Pismarović, Valentina Šoštarčić, Kristina Kljak, Boris Lazarević, Maja Šćepanović

**Affiliations:** 1 Department of Weed Science, Division of Phytomedicine, University of Zagreb Faculty of Agriculture, Zagreb, Croatia; 2 Department of Animal Nutrition, Division of Animal Science, University of Zagreb Faculty of Agriculture, Zagreb, Croatia; 3 Department of Plant Nutrition, Division of Agroecology, University of Zagreb Faculty of Agriculture, Zagreb, Croatia; University of Delhi Department of Environmental Studies, INDIA

## Abstract

With the increasing demand for non-chemical weed control methods, phenolic acids have shown promise due to their natural weed inhibitory potential. In this study, the inhibitory effect of ferulic acid, vanillic acid and *p*-coumaric acid was investigated on *Ambrosia artemisiifolia* L. and the selectivity of *Zea mays* L. against these phenolic acids was tested. The seeds of *A*. *artemisiifolia* and *Z*. *mays* were treated *in vitro* with *three phenolic acids* at doses of 200–600 × 10^−7^ mol and *in vivo* foliar on *A*. *artemisiifolia* and *Z*. *mays* plants. While all phenolic acids had effects on the early growth of *A*. *artemisiifolia*, *p*-coumaric acid significantly reduced the length of radicle and hypocotyl by more than 60% while the effects on *Z*. *mays* were minimal. *In vivo* assessments using chlorophyll fluorescence and multispectral imaging showed selective stress responses in *A*. *artemisiifolia* but not in *Z*. *mays* after foliar application. The *in vitro* results show that *p*-coumaric acid is a promising compound for the control of *A*. *artemisiifolia*. However, these phenolic acids at these doses led to an insufficient reduction in photochemical efficiency. Therefore, these natural compounds need to be combined with other methods of weed control.

## Introduction

In recent decades, following open-world trade and globalization waves, the rate of invasion of alien plants has increased, posing a serious threat to agricultural production and the environment [1]. Perhaps the best example of this is *Ambrosia artemisiifolia* L., common ragweed, a native and widespread weed species in North America that has now spread across many of the European and Asian continents and even Australia [2].

As an invasive plant species, *A*. *artemisiifolia* can inhibit the growth of native plants, destabilize the original ecosystem, and reduce agricultural production [3]. In addition, its pollen can cause a series of allergic reactions and directly affect human health [4]. Consequently, it limits the quality of life of 13.5 million people only in Europe alone [5], with an estimated economic cost of approximately 7.4 billion euros annually [5].

In agriculture, *A*. *artemisiifolia* is listed by the Weed Science Society of America as the ninth most common and troublesome weed in all broadleaf crops, fruits and vegetables in United States and Canada according to 191 survey respondents [6]. Specifically, *A*. *artemisiifolia* is among top five most troublesome weed species in sugar beets, soybean, and cruciferous vegetables, and the most common weed in cucurbits [6]. In parts of Europe [7], the United States [8] and India [9], *A*. *artemisiifolia* was found to be the first broadleaf weed in maize, the world’s leading cereal crop, with more than two hundred million harvested hectares [10]. Studies have shown that maize yields could be reduced by approximately 50% [11] or even 71%, depending on the *A*. *artemisiifolia* density (plants/m^2^) in the maize field [12].

Using mechanical methods alone, such as cultivation and seedbed preparation, may not be sufficient to control *A*. *artemisiifolia* [13]. Therefore, it is suggested that chemical methods should be used for effective control of this weed species [13]. However, the European Green Deal [14] and the Farm to Fork strategy explicitly call for a 50% reduction in pesticide use worldwide by 2030. This implies that chemical solutions are only used as a last resort when other methods of weed control have proved ineffective. Equally important is the realisation that *A*. *artemisiifolia* has developed resistance to herbicides targeting four different sites of action, including ALS inhibitors, auxin mimics, EPSP synthase inhibitors and PPO inhibitors [15], with certain biotypes exhibiting resistance to several classes of herbicides [15].

Natural products, such as essential oils, agricultural byproducts, certain pathogens, and water extracts from many plant species, are gaining increasing attention for their use in weed management [16]. Including cover crops in crop rotation has been shown to effectively control weeds [17] due to the competitive and allelopathic nature of these plants [18]. Consequently, water extracts from allelopathic cover crops have an inhibitory effect on weed growth in plenty of *in vitro* studies [19, 20]. These natural biochemicals present in plants, secondary plant metabolites [21], vary in composition and concentration in different parts of the plant [18] and thus also in the type of natural herbicide (essential oil, water extract, seed meal, etc.) that is used.

Bioactive compounds in cover crops of the Brassicaceae family have been found to have inhibitory effects on germination and early growth of *A*. *artemisiifolia* [22]. Among them, phenolic acids were the most abundant chemical group. Salicylic acid, *p*-hydroxybenzoic acid, cinnamic acid, vanillic acid and ferulic acid are also known to have a strong inhibitory effect on some plant species [23]. In particular, cinnamic acid had strong inhibitory activity in terms of reduction in radicle length and chlorophyll content on *Echinochloa crus-galli* L. [24], same as *p*-coumaric acid, vanillic acid and ferulic acid on *Chenopodium album* L., *Amaranthus retroflexus* L., *Rumex crispus* L., *Plantago lanceolata* L., *Cirsium* sp., *Solanum nigrum* L. [25]. In summary, these compounds have a wide range of effects on cell expansion, membrane permeability, nutrient uptake, photosynthesis, chlorophyll synthesis, and enzyme activity, which may lead to weed suppression [26]. The latest results for ferulic acid and gallic acid show the same effect on weed species in the germination test [27]. In addition, the authors of this study also shed new light on the possible mechanism of action of these phenolic acids, which inhibit the D1 protein, which is crucial for maintaining efficient photosynthesis in plants. However, the *in vivo* effects of these phenolic acids were only observed when they were applied at doses significantly higher than their natural concentrations in plant tissue [24]. Moreover, these effects were obtained by repeated applications over several consecutive days. This method of application and the high doses required are impractical for the use of these compounds as bioherbicides in agricultural production, where consistent and environmentally sustainable practises are essential. Considering the nature of phenolic compounds, and their applicability as bioherbicides, the aim of this study is to select the most effective phenolic acid against *A*. *artemisiifolia* through *in vitro* and *in vivo* trials to further develop it as a complement to other weed control measures, such as tank mixtures with reduced herbicide doses. Therefore, *p*-coumaric acid, ferulic acid and vanillic acid were used in the present *in vitro* and *in vivo* studies with two objectives: I) to investigate the inhibitory and selective potential of these three phenolic acids when used in the same dose to *A*. *artemisiifolia and Z*. *mays*, respectively, *in vitro*, and II) to evaluate the effect of foliar application of these phenolic acids on young *A*. *artemisiifolia* and *Z*. *mays* plants growth with chlorophyll fluorescence and multispectral imaging. Both multispectral and chlorophyll fluorescence imaging are widely used, non-destructive methods for assessing plants stress [28]. These phenolic acids with the same molecular level were, as far as we know, tested for the first time on *A*. *artemisiifolia in vitro* and *in vivo*.

## Results

### Seed bioassay

No statistical difference was observed in reduction of germination and seedling biomass of *A*. *artemisiifolia* and *Z*. *mays* (S1A Table, S2A Table). Furthermore, no significant difference was found between the effect of the applied phenolic acids and doses on the germination dynamics of *A*. *artemisiifolia* compared to the control (S1 File). Therefore, for *A*. *artemisiifolia* and *Z*. *mays* only the results for the reduction of radicle and hypocotyl length and radicle and coleoptile length, respectively, are shown.

#### The effect of phenolic acids on *Ambrosia artemisiifolia L*

**Fig 1:**
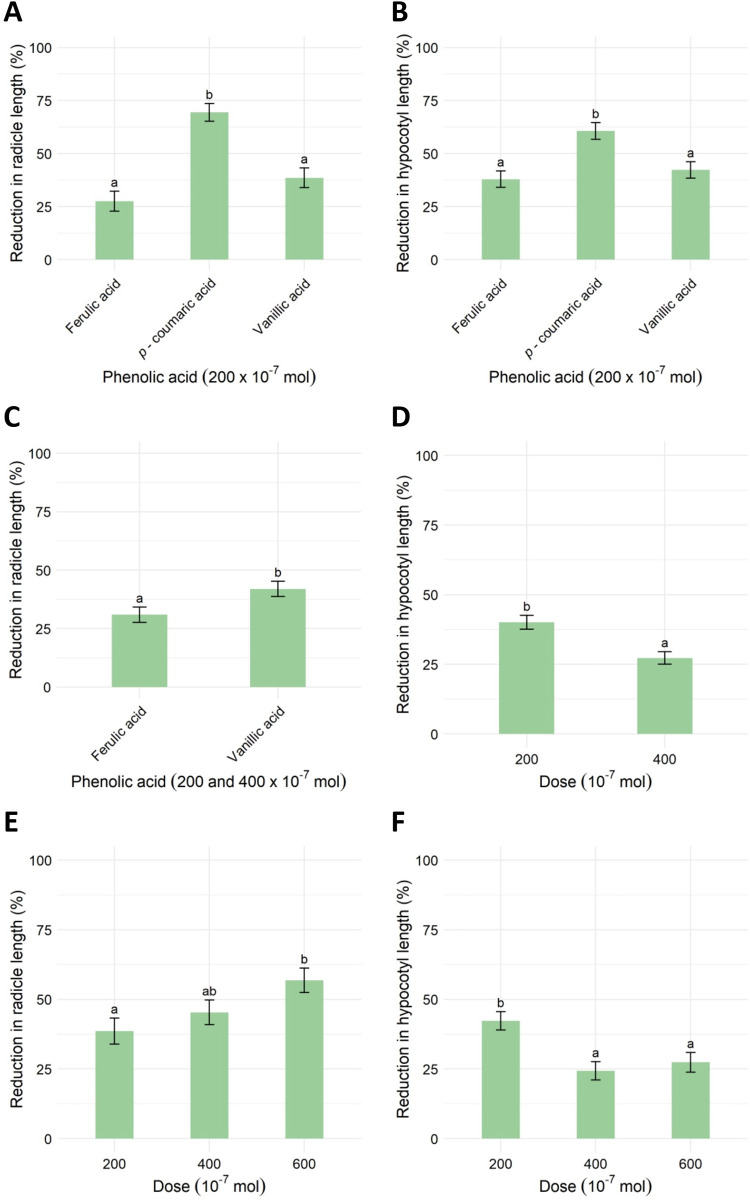
Effect of phenolic acids on the reduction (%) of radicle length (A, C) and hypocotyl length (B), applied doses on radicle length (D) and the effect of applied doses of vanillic acid on radicle and hypocotyl length (E, F) of *Ambrosia artemisiifolia* L. Analysis of variance revealed no difference between the applied doses of ferulic and vanillic acid for radicle length reduction nor between those phenolic acids for hypocotyl length. Thus, C shows the results for the mean values of radicle length reduction averaged over the phenolic acids, and D shows the results for hypocotyl length reduction averaged over the applied doses. The vertical bars represent the estimated marginal means + standard errors. According to Tukey’s test, different lowercase letters represent significant differences (p < 0.05) between the phenolic acids and doses applied.

Differences in the effect of the treatments were found only for the reduction of radicle and hypocotyl length after an applied dose of 200 × 10^−7^ mol (p < 0.001) (S1A Table). The highest reduction in radicle length, 69.41 ± 4.28%, was estimated for *p*-coumaric acid at 200 × 10^−7^ mol (Fig 1A), which was higher than the effects estimated for vanillic acid and ferulic acid, 38.59 ± 4.63% and 27.49 ± 4.69%, respectively.

The reduction in hypocotyl length was again higher when *A*. *artemisiifolia* was treated with *p*-coumaric acid than with vanillic or ferulic acid (Fig 1B), estimated at 60.68 ± 3.91% compared to 42.29 ± 3.91% and 37.19 ± 3.85%, respectively. In contrast, there were no significant differences between phenolic acids in the germination or reduction of seedling weight of *A*. *artemisiifolia* (S1A and S1B Table), although phenolic acids reduced seedling biomass by 45.4% on average.

In the two-way analysis of variance, differences were found in the way phenolic acids (ferulic acid, vanillic acid) affected the reduction in radicle length and the effects of applied doses (200 and 400*10^−7^ mol) on hypocotyl length (S1C Table). Vanillic acid caused a greater reduction in radicle length than ferulic acid (Fig 1C 42.0 ± 3.24% *vs* 30.90 ± 3.25%). Interestingly, hypocotyl length was reduced more by applying a lower dose of phenolic acid than by a higher dose. Again, the weakest effect was observed in germination, where neither ferulic nor vanillic acid caused a reduction of more than 12% at either dose, 200 or 400 × 10^−7^ mol (S1D Table). Furthermore, the second highest extent of reduction in measured parameters in treated *A*. *artemisiifolia* seeds was determined for the fresh weight (S1D Table). On average, fresh weight was reduced by 41.2%.

One-way analysis of variance estimated the differences between the observed effects of applied vanillic acid on radicle and hypocotyl length (S1E Table) of *A*. *artemisiifolia* seedlings.

As expected, the reduction in radicle length was significantly higher at a dose of 600 × 10^−7^ mol (56.9 ± 4.39%) than at the lowest applied dose, 200 × 10^−7^ mol (38.6 ± 4.71%; Fig 1E). In contrast, hypocotyl length was reduced the most by vanillic acid applied at the lowest dose (200 × 10^−7^ mol; Fig 1F), by 42.3% ± 3.26 in contrast to 24.3% ± 3.31 or 27.4% ± 3.54 at doses 400 or 600 × 10^−7^ mol, respectively. The extent of the fresh weight reduction was again the second highest in comparison with the effects of vanillic acid on other measured parameters. The fresh weight was reduced by 46.3% on average (S1F Table). Vanillic acid had the weakest effect on the germination of *A*. *artemisiifolia* seeds. The average reduction in germination was estimated at 11.96%.

Analysis using the non-parametric time-to-event model and the permutation test revealed that there were no significant differences in the germination dynamics of *A*. *artemisiifolia* seeds treated with the three phenolic acids at doses of 200 to 600 x 10^−7^ mol (S1 File). Therefore, these phenolic acids at these doses had no effect on the germination rate of *A*. *artemisiifolia* in this study.

#### The effect of phenolic acids on *Zea mays L.*

**Fig 2:**
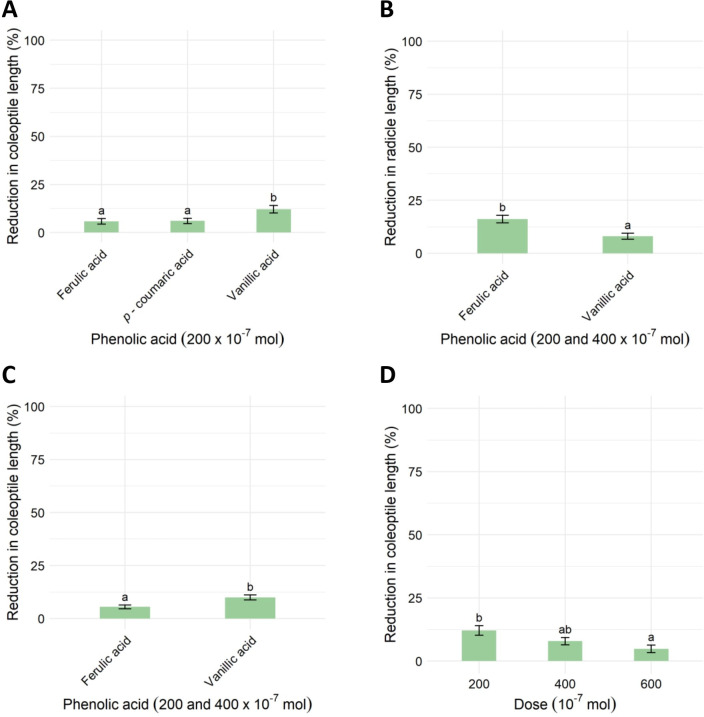
The effect of phenolic acids on the reduction (%) of coleoptile length (A, C), radicle length (B) and the effect of the applied doses of vanillic acid on coleoptile length (D) of *Zea mays* L. Analysis of variance revealed no difference between the applied doses of ferulic and vanillic acid for radicle/coleoptile length reduction. Therefore, B shows the results for the mean values of radicle length reduction averaged over the phenolic acids, and C shows the results for coleoptile length reduction averaged over the applied phenolic acids. The vertical bars represent the estimated marginal means + standard errors. According to Tukey’s test, different lowercase letters represent significant differences (p < 0.05) between the phenolic acids and doses applied.

Analysis of variance of the obtained data from germination and measured early growth parameters in *Zea mays* L. seedlings found differences in the effect caused by applied phenolic acids at a dose of 200 × 10^−7^ mol only for coleoptile length (S2A Table). Coleoptile length was reduced by 12.12 ± 1.95% by vanillic acid, while ferulic and *p*-coumaric acid caused two-fold lower reductions, namely, 5.83 ± 1.40% and 6.07 ± 1.37%, respectively (Fig 2A).

There was no significant difference between applied phenolic acids in the reduction of germination, seedling weight, and radicle length of *Z*. *mays*. However, it should be noted that from the determined parameters, the applied phenolic acids exhibited the highest reduction in radicle length, where the radicle length was reduced by 14.3% on average (S2B Table). Significant differences in the reduction of radicle and coleoptile length of *Zea mays* L. were found between ferulic acid and vanillic acid at doses of 200 and 400 × 10^−7^ mol (S2C Table, Fig 2B and 2C). The radicle length was reduced by 16.18 ± 1.78% by ferulic acid, while vanillic acid caused half as much reduction, 8.04 ± 1.46%, regardless of the applied dose. In contrast, the length of the coleoptile was reduced more by vanillic acid (9.99 ± 1.18%) than by ferulic acid (5.51 ± 0.94%), regardless of the applied dose. In general, the lowest extent of reduction by ferulic and vanillic acid was found for the germination parameter (S1D Table). The germination was reduced by 1.66% on average. In contrast to germination, the fresh biomass weight of *Z*. *mays* seedlings was reduced by 4.26% on average.

The analysis of variance estimated differences only for the observed effects of applied doses of vanillic acid on the reduction (%) of coleoptile length of *Z*. *mays* seedlings (S1E Table, Fig 2D). Similar to the effect on hypocotyl length of *A*. *artemisiifolia*, the highest effect of vanillic acid was at the dose of 200 × 10^−7^ mol, 12.12 ± 1.91%, while the dose 600 × 10^−7^ mol caused a notably lower reduction in comparison, 4.80 ± 1.52%. Furthermore, the highest extent of reduction caused by vanillic acid was determined for the radicle length of the *Z*. *mays* seedlings, with a 9.01% reduction on average (S2F Table). The fresh weight of *Z*. *mays* seedlings was reduced by 3.83% on average, while germination was almost not affected at all, with a 0.44% reduction on average (S2F Table).

### Growth chamber bioassay

The interaction between phenolic acids and day of measurement was found for almost all measured and calculated traits (S3A and S3B Table). Analysing the variable clustering analysis effectively reduced the dimensionality of our dataset. From an initial set of 15 chlorophyll fluorescence variables and 12 multispectral variables, the hierarchical clustering algorithm grouped them into four (Table 1) and three distinct clusters (Table 2), respectively.

**Table 1:** Results of the cluster analysis for traits measured and calculated by chlorophyll fluorescence imaging.

Cluster	No. of Members	Most Representative Variable	Cluster Proportion of Variation Explained	Total Proportion of Variation Explained
1	6	NPQ	0.827	0.355
2	4	q_P_	0.698	0.199
3	2	ф_no_	0.86	0.123
4	2	F_m_	0.765	0.109
**Cluster**	**Members**	**RSquare with Own Cluster**	**RSquare with Next Closest**	**1-RSquare Ratio**
1	NPQ	0.926	0.211	0.093
1	ф_npq_	0.88	0.314	0.175
1	q_N_	0.861	0.322	0.205
1	rETR	0.815	0.116	0.209
1	F_q’_/F_m’_	0.744	0.099	0.284
1	F_m’_	0.737	0.485	0.51
2	q_P_	0.869	0.128	0.15
2	q_L_	0.51	0.052	0.517
2	F_v_/F_m_	0.665	0.377	0.538
2	F_0_	0.749	0.623	0.666
3	ф_no_	0.86	0.14	0.162
3	F_s’_	0.86	0.49	0.274
4	F_m_	0.765	0.142	0.274
4	F_0’_	0.765	0.577	0.556

**Table 2:** Results of the cluster analysis for traits measured and calculated by multispectral imaging.

Cluster	No. of Members	Most Representative Variable	Cluster Proportion of Variation Explained	Total Proportion of Variation Explained
1	8	SpcGrn	0.791	0.527
2	3	Blue	0.69	0.172
3	1	Nir	1	0.083
**Cluster**	**Members**	**RSquare with Own Cluster**	**RSquare with Next Closest**	**1-RSquare Ratio**
1	SpcGrn	0.912	0.003	0.088
1	Value	0.912	0.005	0.088
1	Green	0.911	0.022	0.091
1	Red	0.876	0.078	0.135
1	AriIdx	0.72	0.071	0.302
1	FarRed	0.675	0.14	0.378
1	ChlIdx	0.698	0.297	0.429
1	NDVI	0.623	0.352	0.581
2	Blue	0.823	0.158	0.21
2	Saturation	0.73	0.25	0.36
2	Hue	0.516	0.064	0.517
3	Nir	1	0.07	0

Within each cluster, the variable with the highest average correlation was selected as the most representative variable. This selection process resulted in a reduced set of representative variables that covered most of the variation in the original data. The clusters exhibited strong internal cohesion, as indicated by the high intra-cluster correlation coefficients (Table 1 and Table 2).

The dimension reduction facilitated by variable clustering not only simplified the data set, but also improved the interpretability of our models. The resulting representative variables and cluster components were used in the subsequent analyses.

In addition to the variables selected through the cluster variable analysis (NPQ, q_P_, ф_no,_ F_m_, SpcGrn, Blue, Nir), the Normalized Difference Vegetation Index (NDVI) was included in the subsequent analysis as it has proven to be efficient and is widely used in the assessment of plant health and vigour.

The inclusion of the NDVI was justified by the fact that it is recognised in the scientific community as a reliable and non-destructive measure of vegetation health that has been extensively validated in numerous studies. A repeated measures ANOVA was again performed, but with the "SLICE" option for the day of measurement (T1-T7), which revealed a significant difference between the phenolic acids and the control treatment within days T1, T2 and T7 (S4). Therefore, the observed effects of phenolic acids on *A*. *artemisiifolia* are detailed further only for the representative measured and calculated traits with significant effects, F_m_, q_P_, NPQ and NDVI.

**Fig 3:**
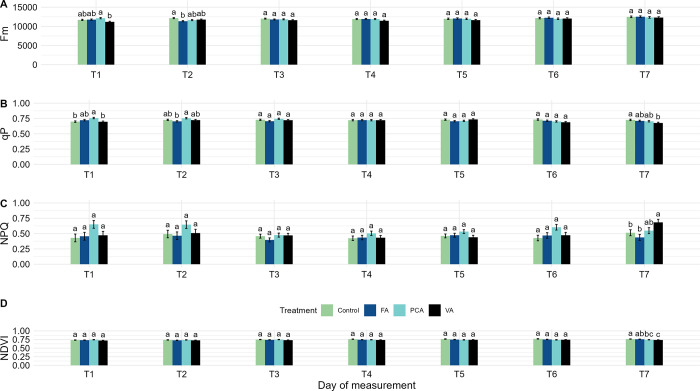
The effect of phenolic acids on the selected chlorophyll fluorescence and multispectral traits of young *A*. *artemisiifolia* plants. The effect of phenolic acids, *p*-coumaric acid (PCA), ferulic acid (FA), vanillic acid (VA) and distilled water as a control treatment (Control), was measured by chlorophyll fluorescence and multispectral imaging and analysed by repeated measures analysis of variance by “SLICE” option. The vertical bars represent the mean values + standard errors. According to Tukey’s test, different lowercase letters represent significant differences (p < 0.05) between the treatments. Selected traits of chlorophyll fluorescence where a difference was observed between treatments were maximum fluorescence—F_m_ (A), coefficient of photochemical quenching—q_P_ (B), non-photochemical quenching—NPQ (C), while of the multispectral traits only normalised difference vegetation index—NDVI (D) showed a significant difference between treatments. The treatments were applied twice, on T1 and T3, using a hand sprayer. The effect of the treatments was measured on seven consecutive days (T1-T7), with T1 and T3 measured after application and absorption of phenolic acids.

On the first day of measurement (T1), after the first application of phenolic acids, and on the second day 24 hours after application (T2), earliest effects were found only for F_m_ and q_P_ (Fig 3A). On day T1, *p*-coumaric acid caused significant increase Fm and qP, while vanillic acid decreased those traits, suggesting that *p*-coumaric acid possibly increased the photosynthetic efficiency or capacity of the treated plants. However, a difference between the treated plants and the control plants was only found for q_P_ on T1. On the day after application (T2), the highest Fm was observed in the control plants while ferulic acid caused decrease in Fm (Fig 3B). However, the plants treated with *p*-coumaric acid showed the same trend as at T1, namely the highest values of q_P_, in contrast to ferulic acid, which decreased at T2. There were no significant differences in the effect of phenolic acids for days T3-T6 on the F_m_ q_P_, NPQ, and NDVI (Fig 3A–3D).

**Fig 4:**
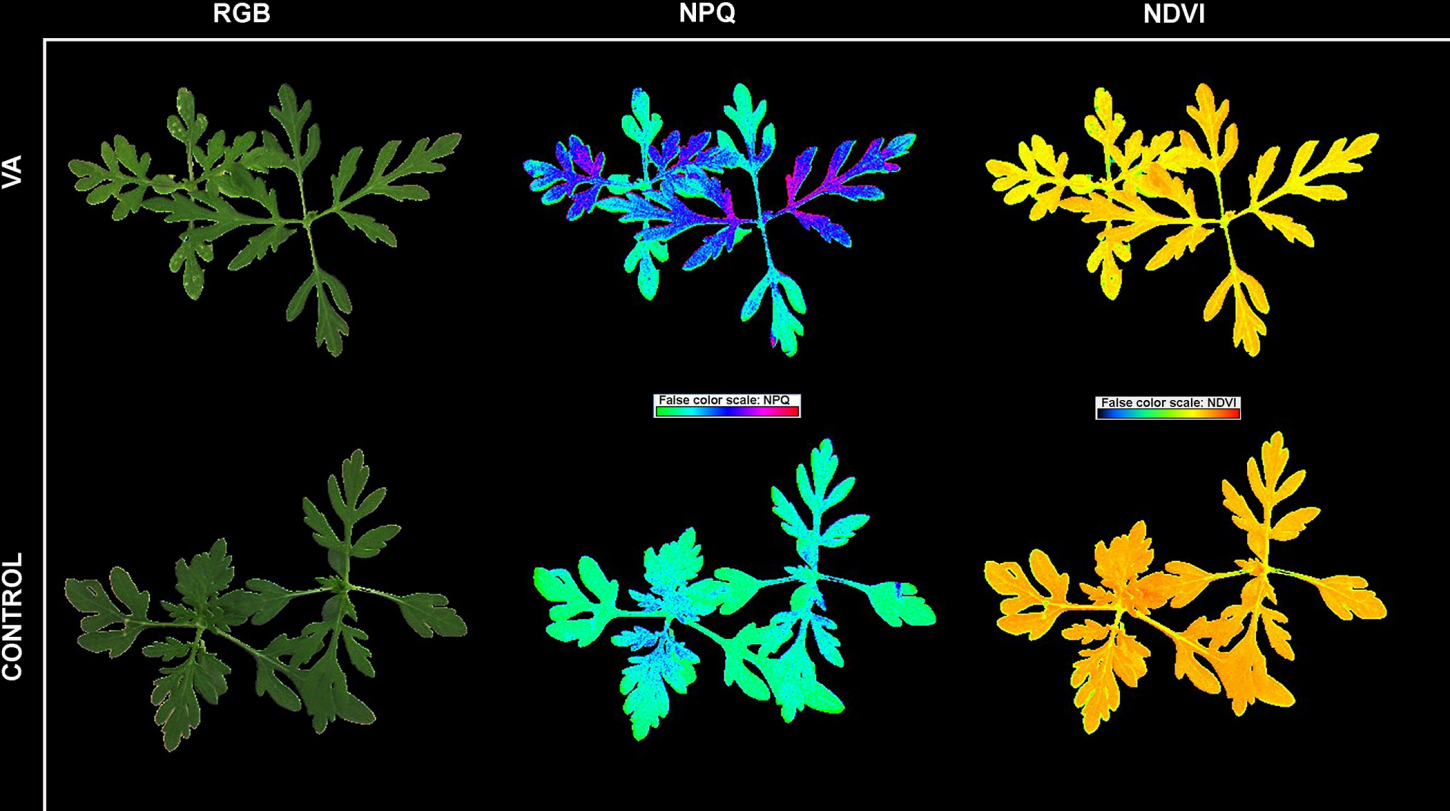
*Ambrosia artemisiifolia* L. color and pseudo-color images. Figure shows RGB (visual impression), non-photochemical quenching (NPQ) and normalised difference vegetation index (NDVI) images of young *A*. *artemisiifolia* plants 7 days after first application (T7) of vanillic acid (VA) and control plants sprayed with distilled water (CONTROL).

The multispectral variables showed differences only for NDVI at T7, while chlorophyll fluorescence showed differences only for q_P_ and NPQ. Analysing the obtained NPQ, q_P_ and NDVI at T7 revealed significant differences between the vanillic acid-treated plants and the control plants. In particular, the increase of NPQ and decrease of q_P_ of the vanillic acid-treated plants indicate that the treated plants are under stress compared to the control plants, which showed opposite for these measured traits. The differences could not be detected visually or by comparing the RGB images of the treated plants. However, when using chlorophyll fluorescence and multispectral channels (NDVI and NPQ), the difference in the response of treated and untreated plants was evident (Fig 4). The increased NDVI for the control plants and decreased for vanillic acid are consistent with the results of NPQ and q_P_. Specifically, based on the measurements at T7 4 days after the second application of phenolic acids, a significant effect on the young *A*. *artemisiifolia* plants was observed in the vanillic acid-treated plants, while the measurements of the control plants can be interpreted as those of plants with normal health and vigour.

One-way ANOVA revealed no significant effect of ferulic acid on the selected multispectral and chlorophyl fluorescence traits on *Z*. *mays* plants (S5). Furthermore, the plants were monitored daily for the next three weeks to observe potential symptoms of phytotoxicity. However, there was no delayed phytotoxic response to foliar application of ferulic acid on *Z*. *mays* plants indicating selective behaviour to this compound.

## Discussion

It is of great importance to find new ways to control weed growth and distribution, especially those that are environmentally friendly, practical, and an alternative to chemical control. This is especially important for herbicide-resistant populations as well as for aggressive invasive plant species that are harmful to autochthonous flora, such as *A*. *artemisiifolia*. In this study, the emphasis was on determining the herbicidal effect of inhibitory allelochemicals on *A*. *artemisiifolia* and the selectivity of *Z*. *mays* for these allelochemicals. Although vanillic acid, ferulic acid, and *p*-coumaric acid, have already been tested individually [29], it is important to test their efficacy at the same molecular level, i.e., at the same dose, and to investigate their effect on physiological processes after foliar application on young *A*. *artemisiifolia* and *Z*. *mays* plants.

The three phenolic acids (ferulic, vanillic, and *p*-coumaric) had a greater impact *in vitro* on the radicle or hypocotyl/coleoptile length of both tested plants (*A*. *artemisiifolia* and *Z*. *mays*) than on the germination or fresh weight of seedlings. It was expected that the radicle length would be the most sensitive parameter as it emerges first from the seed and is in direct contact with the solution longer than the hypocotyl or coleoptile. The fact that roots are early germination parameter the most sensitive to the inhibitory effects of phenolic acids is already explained by the effect of phenolic acid on the inhibition of cell division and the increased thickness of seminal roots in direct contact with allelochemicals [24] which is consistent with another study in which the radicle was also found to be the most sensitive parameter of six different weed species [25]. In particular, the mixture of ferulic and vanillic acids caused root cell death in the weed species *Pinellia ternata* (Thunb.) Makino, which is further explained by the overaccumulation of H_2_O_2_ and O^-2^ in the roots [30]. In addition to the reduction in radicle length observed in the present study, some phenolic acids, such as vanillic and ferulic acids, can also cause phytotoxic symptoms, such as brown staining of the root apices [31]. It was found that in soybean roots, these three phenolic acids influence phosphate and methionine uptake and their incorporation into nucleic acids and proteins. Thus, this kind of interference is one of the main mechanisms by which phenolic acids influence plant growth [32]. In addition, *p*-coumaric and ferulic acid have been studied to have a similar mode of action to herbicides that inhibit acetolactate synthesis [33], resulting in internal accumulation of these acids in treated plants, growth arrest, accumulation of carbohydrates in roots and leaves, accumulation of quinate in leaves and induction of ethanolic fermentation. Furthermore, *p*-coumaric acid increased lignin content in soybean roots [34], which is known to be the response after contact with allelochemicals [35]. *P*-coumaric and ferulic acid led to excessive production of lignin and its main monomers, stiffening the cell wall and thus preventing root growth [36]. The latest findings show that gallic acid and ferulic acid inhibit the function of a D1 protein in *Sinapis arvensis* L., *Lolium multiflorum* Lam. and *Parthenium hysterophorus* L., which is a crucial component of Photosystem II (PSII) [27] and whose inhibition negatively affects the photosynthetic activity of the weeds. This finding is reflected in our study, in which ferulic acid decreased F_m_ and q_P_ on T2 (Fig 3A). Nevertheless, it is currently difficult to say whether there is a single site of action or whether the phenolic acids have multiple sites of action (26). In addition, it was found that the way plants absorb water extracts can influence the effectiveness of phenolic acids. For example, significant difference was found in the effect of water extracts of *Eucalyptus globulus* Labill on adult *Lactuca sativa* L. plants after foliar application and after irrigation of the plants with the same treatments [37]. The water extracts contained two derivatives of *p*-coumaric acid and the effect was assessed by chlorophyll fluorescence imaging and protein content. After foliar application, the treatments caused a reduction in aerial and root biomass and increased the dry weight/fresh weight ratio of *L*. *sativa* adult plants. In contrast, when the plants were watered with water extracts, the protein content and chlorophyll concentration of the plants decreased, indicating different sites of action depending on the way of uptake of the allelochemicals. This could explain why *p*-coumaric acid has caused strong inhibition of *A*. *artemisiifolia* radicle length *in vitro*, and weak effect after foliar application in our study (Figs 3 and 4). Although certain differences were found in comparison to the control plants, the results of the *in vivo* experiments indicate that the phenolic acids tested at these doses do not have a significant inhibitory effect. In particular, *p*-coumaric acid showed a stronger effect on *A*. *artemisiifolia* than vanillic or ferulic acid after the first foliar application Both q_P_ and F_m_ were highest in the *p*-coumaric acid treated plants. This indicates that the plants are very efficient at both capturing light energy (F_m_) and utilizing that captured energy for photochemistry (q_P_). However, this effect was not observed from T3 to T7. In contrast, the effect of vanillic acid reappeared 7 days after the first foliar application. Both the low F_m_ and q_P_ values at T1 indicate damage to the photosynthetic apparatus, which reappeared at T7 and is reflected in a high NPQ and a low NDVI. It is possible that the plants had an immediate stress response that temporarily disrupted photosynthesis (T1) while they acclimatised over the next six days and activated compensatory mechanisms that restored normal photosynthetic function. On the seventh day, the plants possibly experienced delayed effects or a cumulative effect of the vanillic acid mode of action along with the consequences of overcompensation after the first foliar application. re. This is in line with the results of Anwar et al. [27], who showed that the effective use of ferulic acid *in vivo* requires much higher doses and several consecutive applications. Although these natural bioactive compounds alone cannot completely inhibit the germination and growth of *A*. *artemisiifolia* [29], they could be useful in control of this weed species by providing an additional means of control. This would be particularly effective if they were combined with a reduced dose of a specific herbicide, which should be tested in different crops.

The observed stronger effect of phenolic acids on reducing the growth of *A*. *artemisifolia* compared to *Z*. *mays* in our study indicates a potential benefit in weed management strategies. This selective herbicidal effect is promising for targeted weed control and may allow the suppression of weed species without significantly affecting crop growth. Indeed, the radicle length of *Z*. *mays* was reduced by a maximum of 16% when doses of 200 or 400 × 10^−7^ mol of ferulic acid were applied. Bashar et al. [38] investigated the effect of *P*. *hysterophorus* methanolic extracts on selected plants and weeds and found maize to be less susceptible to these extracts than dicotyledon crops and weeds. *p*-coumaric acid, ferulic acid and vanillic acid were all identified in significant concentrations in those extracts and are considered to be responsible for the inhibitory effect on the selected plants and weeds [38]. Krogmeier and Bremner [39] tested nine phenolic acids on the germination and early growth of six cultivated plants, including *Z*. *mays*. After the application of *p*-coumaric acid, ferulic acid and vanillic acid, the germination of *Z*. *mays* was not affected, whereas the germination of alfalfa seeds was reduced by up to 27%. However, in their greenhouse experiments, no adverse effects on alfalfa emergence, seedling growth or early plant development were observed for any of the phenolic acids tested. Our experiment yielded similar results, as *Z*. *mays* treated with ferulic acid showed no phytotoxic symptoms during the entire four weeks of the in vivo experiment. Furthermore, neither subtle damage nor changes in photochemical efficiency were detected, as shown by the multispectral and chlorophyll fluorescence analyses (S5 Table). These results indicate that certain phenolic acids, including ferulic acid, do not pose significant phytotoxic risks to *Z*. *mays* under similar experimental conditions.

However, some studies suggest that the effect of phenolic acids on germination and development may also depend on the maize hybrid [40] which should be further investigated. Effective weed control by combining allelopathic water extracts and reduced herbicide doses has been demonstrated in the literature [41, 42].

In this study, the phenolic acids were individually tested to compare the efficacy of each at the same dose, knowing the complexity of explaining interactions between various bioactive compounds and their effect on evaluation parameters. This was a first step in the search for new alternatives for sustainable chemical control. The results of this study indicate that while phenolic acids have potential as bioherbicides, their practical application requires overcoming challenges related to effective dosing and delivery. The selective inhibitory effect on *A*. *artemisiifolia* compared to *Z*. *mays* supports their use as part of integrated weed control strategies, especially in combination with reduced doses of conventional herbicides. In addition, future research should focus more on developing optimised application methods for field conditions. Of course, the long-term effects and potential for the development of resistance of weeds to bioherbicides, as with synthetic herbicides, should also be explored. Since phenolic acids can affect crop yield and plant growth, the effects of phenolic acids on beneficial soil microbiota and fauna must also be investigated. These steps will be crucial for the further development of sustainable weed control strategies and the management of herbicide-resistant populations such as *A*. *artemisiifolia* [15].

Indeed, *p*-coumaric acid, which was found to have the highest herbicidal potential against *A*. *artemisiifolia* in our study, was found to inhibit the germination and growth of two herbicide-resistant biotypes of *Lolium rigidum* Gaudin [43]. Research into alternative methods, such as the use of phenolic acids, remains an important pathway in the context of sustainable weed control.

## Materials and methods

Phenolic acids were applied to *A*. *artemisiifolia* and *Z*. *mays* seeds *in vitro* to estimate the inhibitory potential of ferulic acid, vanillic acid, and *p*-coumaric acid through germination dynamics and reduction of germination percentage, hypocotyl/coleoptile length, radicle length and seedling biomass of *A*. *artemisiifolia* and *Z*. *mays*.

The effect of foliar applied phenolic acids was investigated on young *A*. *artemisiifolia* plants (BBCH 12) during 7 consecutive days after first application, by chlorophyll fluorescence and multispectral imaging. The effects of ferulic acid on *Z*. *mays* was analysed by these methods on 7th day after foliar application.

### Seed material

In October 2018, mature seeds of *A*. *artemisiifolia* were collected at the Šašinovec Experimental Station (45°51’05”.2 N, 16°10’34”.1 E) at the University of Zagreb Faculty of Agriculture, Croatia. The seeds were cleaned and stored in paper bags at 4°C. Only seeds of uniform size and colour that showed no visible signs of insect feeding were selected for the experiment. Germination tests of the seeds of the selected population showed that > 70% of the tested seeds germinated. The maize seeds used for the experiment (Syngenta, SY Carioca) were also checked and selected to ensure the homogeneity of the sample.

### Seed bioassay

The reference standards for the three phenolic acids used were obtained from Sigma–Aldrich (Steinheim, Germany): ferulic acid (trans-4-hydroxy-3-methoxycinnamic acid), vanillic acid (4-hydroxy-3-methoxybenzoic acid) and *p*-coumaric acid (trans-4-hydroxycinnamic acid). These phenolic acids were dissolved individually in distilled water and sonicated at 35 kHz and 80°C (Sonorex TK 52, Bandelin, Germany) to obtain homogeneous solutions at different doses. Tested phenolic acids differ in their solubility in water [44]. Therefore, vanillic acid was dissolved in doses of 200, 400, and 600 × 10^−7^ mol, ferulic acid was dissolved in doses of 200 and 400 × 10^−7^ mol, and *p*-coumaric acid was dissolved in a dose of 200 × 10^−7^ mol. These doses were calculated based on the natural occurrence of these phenolic acids in Brassicaceae (*Sinapis alba* L., *Raphanus sativus* var. *oleiformis*, and *Camelina sativa* (L.) Crantz) plant species sown as cover crops [22]. The dose of 200 × 10^−7^ mol corresponds to approximately 2.5, 4 and 5 times the dose of ferulic acid, vanillic acid, and *p*-coumaric acid, respectively, naturally occurring in these cover crops.

For each of the treatments, a total of 50 *A*. *artemisiifolia* seeds were placed on filter paper in a Petri dish (90 mm diameter), after which 4 ml of a single phenolic acid was added at a fixed dose. Distilled water (4 ml per Petri dish) was added as a control treatment. Each treatment (including the control) with each dose was repeated four times, and the whole experiment was repeated twice. The dishes were placed in a climate chamber (HPP 108, Memmert, Schwabach, Germany) under the following conditions: photoperiod, 12 h/12 h; day temperature, 25°C; night temperature, 15°C; humidity, 70%; and light intensity, 40–50 μmol/m2 (LED light). All dishes were hermetically sealed with Parafilm to prevent evaporation.

After 10 days, the germination and early growth, hypocotyl length, radicle length, and fresh weight of the 10 *A*. *artemisiifolia* seedlings were measured per Petri dish. The seeds were classified as germinating if their radicle length was >1 mm. The same methodology was used for the seeds of *Z*. *mays* with minor modifications. Twenty-five *Z*. *mays* seeds were treated with 5 ml of each phenolic acid or distilled water in Petri dishes 12 mm in diameter. The germination, coleoptile length, radicle length and fresh weight of the *Z*. *mays* seedlings were measured after 7 days. The fresh weight of the seedlings was weighed on an analytical balance (MS105DU, Mettler Toledo, Greifensee, Switzerland).

The percentage of inhibition, reflected through the length of the radicle, coleoptile or hypocotyl, and seedling biomass were calculated using Abbot’s reduction coefficient formula [45]:

$$\%inhibition=\left[\frac{Xc-Xt}{Xc}\right]x100$$

where Xc is the length of the coleoptile/hypocotyl or biomass weight of the control seedlings and Xt is the length of the coleoptile/hypocotyl or biomass weight of the seedlings treated with phenolic acids.

For the bioassay on the germination dynamics of *A*. *artemisiifolia*, the experiment was set up as described. The germinated seeds were checked daily for 41 day until no germinated seeds were found for ten consecutive days. The germinated seed data obtained were used to generate a germination dynamics for each phenolic acid at each dose and for the control. The daily germination data were used to determine the germination dynamics (germination rate) within the analysed period.

### Growth chamber bioassay, chlorophyll fluorescence and multispectral imaging

To obtain sufficiently uniform plant material, over 4000 *A*. *artemisiifolia* seeds were sown in germination trays (containers). From sowing until the end of the experiment, *A*. *artemisiifolia* was grown in a growth chamber under the following conditions: 25/15°C, 12/12 h day/night regime, 70% relative humidity and 250 μmol m^–2^ s^–1^ photosynthetic photon flux density (PPFD) provided by Valoya L35, NS12 spectrum LED lights (Valoya Oy, Helsinki, Finland). When the first two leaves were observed on the emerged plants, 40 equally developed plants were selected and transplanted into 0.18 L plastic pots filled with the substrate mixture of Potgrond H (Klasmann-Deilmann GmbH) and the perlite Agroperl-G (Agroperl®) in a ratio of 80:20. The plants were irrigated on alternate days. Twenty-four hours after transplanting, the plants were treated with phenolic acids at the highest doses: 200 × 10^−7^ mol *p*-coumaric acid, 400 × 10^−7^ mol ferulic acid and 600 × 10^−7^ mol vanillic acid, The first foliar treatment was applied one day after the development of the first true leaves (T1), and the second treatment followed 48 hours later (T3). The phenolic acids were applied foliar using a TLC sprayer (CAMAG®, Muttenz, Switzerland), while the control plants were sprayed with distilled water.

The experiment was conducted as a complete block randomized design, with 12 plants per treatment. Each phenolic acid treatment was replicated six times, with each replication consisting of a plastic pot containing two plants. Chlorophyll and multispectral images were taken on seven consecutive days (T1-T7), starting from the day of the first treatment application (T1).

Analysis was performed using CropReporterTM (PhenoVation B.V., Wageningen, The Netherlands). Each day, the plants were dark-adapted for 30 minutes before imaging. Red LED light at 4500 μmol m^−2^ s^−1^ was used for chlorophyll fluorescence excitation. Minimum chlorophyll fluorescence (F_0_) was measured after 20 μs, maximum chlorophyll fluorescence (F_m_) after saturation (800 ms). Subsequently, the plants were acclimated to light under 250 μmol m^−2^ s^−1^ LEDs for 5 min. Red LED light at 4500 μmol m^−2^ s^−1^ was again used to excite chlorophyll fluorescence. At the beginning of the saturation pulse, the steady-state fluorescence (F_s′_) was measured and the maximum chlorophyll fluorescence (F_m′_) at saturation (800 ms). Afterwards, far-red light was switched on and the minimum fluorescence yield of the illuminated plant (F_0′_) was estimated. From the described measurements, several fluorescence parameters were automatically calculated: (i) maximum efficiency of PSII (F_v_/F_m_) [46]; (ii) the effective quantum yield of PSII (F_q′_/F_m′_) [47]; (iii) electron transport rate (ETR) [47]; (iv) non-photochemical quenching (NPQ) [48]; (v) coefficient of photochemical quenching (q_P_) [49]; (vi) coefficient of non-photochemical quenching (q_N_) [49]; (vii) estimation of ‘open’ reaction centres based on a lake model (q_L_) [50]; (vii) quantum yield of non-regulated non-photochemical energy loss in PSII (ɸ_nq_) [51]; and (ix) quantum yield of regulated non-photochemical energy loss in PSII (ɸ_npq_) [51].

Multispectral imaging was performed under 250 μmol m^−2^ s^−1^ LEDs. Reflectance images were collected at red (peak wavelength 640 nm), green (peak wavelength 500 nm), blue (peak wavelength 475 nm), specific green (peak wavelength 510–590 nm), far red (peak wavelength 710 nm), NIR (peak wavelength 769 nm), specific for chlorophyll at 730 nm and specific for anthocyanins at 550 nm. From the collected spectral reflectance data, different vegetation indices were calculated: chlorophyll index (CHI) [52] and anthocyanin index (ARI) [53], normalised differential vegetation index (NDVI) [54], hue (0–360°), saturation (SAT), and value (VAL).

For the bioassay in the growth chamber, *Z*. *mays* seeds of the same type and manufacturer were selected that had the same size and morphology as those used in the seed bioassay. Ten maize seeds (per pot) were sown 4 cm deep from the surface in growing pots (20 × 20 cm) filled with the same substrate mixture as for *A*. *artemisiifolia*. After sowing, the pots were placed in the growth chamber at the optimum temperature for the growth of *Z*. *mays*: 27/20°C with 70% humidity and a photoperiod of 14 h/10 h (day/night). The *Z*. *mays* seedlings were continuously monitored throughout the experiment. After germination, until the second leaf growth stage (BBCH 12), the young *Z*. *mays* plants were thinned from 10 plants to final three left in one pot. The plants were selected so that the plants in the pot remained as uniform as possible at the time of application, as the symptoms of phytotoxicity were to be monitored after application. Based on the results of the *in vitro* assay and the highest reduction in growth caused by ferulic acid, only ferulic acid at a dose of 400*10^−7^ mol was used for foliar application on *Z*. *mays*. The treatment was carried out in three replicates and the control plants were sprayed with distilled water. The effect of ferulic acid on *Z*. *mays* young plants was analysed on the seventh day after application using the same methods as described above. Thereafter, *Z*. *mays* was further monitored over the following three weeks to observe a possible delayed response. CropReporterTM and described imaging are explained in more detail by Lazarević et al. [28].

### Statistical analysis

#### Seed bioassay

The laboratory experiment was performed twice with four replicates each time. No differences were identified between the experiments by t test. The data were combined for analysis and subjected to graphical inspections to assess the normality of distribution and homogeneity of variances. Data obtained from germination and measured early growth parameters (radicle length, hypocotyl/coleoptile length, seedling biomass) in the seeds of *A*. *artemisiifolia* and *Z*. *mays* treated with ferulic and vanillic acids at doses of 200 and 400 × 10^−7^ mol were processed using two-way analysis of variance with phenolic acids, applied dose and their interaction as fixed effects and repetition as a blocking factor. The rest of the data, for dose 200 × 10^−7^ mol (all three phenolic acids) and vanillic acid in doses 200, 400, and 600 × 10^−7^ mol, were processed using one-way analysis of variance with dose as a fixed effect and repetition as blocking effect. The difference between the estimated marginal means [55] of the inhibition percentage was checked with the post hoc Tukey HSD test. Germination dynamics were analysed using the non-parametric time-to-event model described by Onofri et al. [56]. The permutation test based on a Cramer-von-Mises type distance [57] was used to detect differences between the germination dynamics of the treated seeds of *A*. *artemisiifolia*. Data were analysed in R software and environment [58].

#### Chlorophyll fluorescence and multispectral analysis

Repeated measures analysis of variance (ANOVA) was performed in JMP^®^ (Version <x>. SAS Institute Inc., Cary, NC, 1989–2023) on data obtained for *A*. *artemisiifolia* L.

Selected variables from the cluster variable analysis were further analysed using repeated measures ANOVA, with treatments (n = 4) as fixed effects, measurement time (MT) (n = 7) as a repeated measure, and pots (n = 6) as subjects within treatments for *A*. *artemisiifolia*. Covariance was modelled according to residual log-likelihood and the Akaike information criterion. Tukey’s Honest Significant Difference post hoc test was performed for partitioned F-tests (SLICE option) to examine the significance of treatment differences within each measurement time point. A one-way ANOVA was performed on the before selected traits for *Z*. *mays*.

## Supporting information

S1 Table Analysis of variance (ANOVA) and reduction (%) of germination and early growth parameters in *Ambrosia artemisiifolia* L. seedlings by phenolic acids. Table A: ANOVA for the reduction (%) of measured early growth parameters and germination in *Ambrosia artemisiifolia* L. by phenolic acids at a dose of 200 × 10−7 mol. Table B: The reduction (%) of germination and measured early growth parameters in *Ambrosia artemisiifolia* L. seedlings by phenolic acids at a dose of 200 × 10−7 mol. Table C: Two-way ANOVA for the reduction (%) of measured early growth parameters and germination in *Ambrosia artemisiifolia* L. by vanillic and ferulic acids at doses of 200 and 400 × 10−7 mol. Table D: The reduction (%) of germination and measured early growth parameters in *Ambrosia artemisiifolia* L. seedlings by ferulic and vanillic acids at doses of 200 and 400 × 10−7 mol. Table E: ANOVA for the reduction (%) of germination and measured early growth parameters in *Ambrosia artemisiifolia* L. by vanillic acid at doses of 200, 400 and 600 × 10−7 mol. Table F: The reduction (%) of germination and measured early growth parameters in *Ambrosia artemisiifolia* L. seedlings by vanillic acid applied in doses of 200, 400 and 600 × 10−7 mol.
(PDF)

S2 Table Analysis of variance (ANOVA) and reduction (%) of germination and early growth parameters in *Zea mays* L. seedlings by phenolic acids. Table A: ANOVA for the reduction (%) of measured early growth parameters and germination in *Zea mays* L. by phenolic acids at a dose of 200 × 10^−7^ mol. Table B: The reduction (%) of germination and measured early growth parameters in *Zea mays* L. seedlings by phenolic acids at a dose of 200 × 10^−7^ mol. Table C: Two-way ANOVA for the reduction (%) of measured early growth parameters and germination in *Zea mays* L. by vanillic and ferulic acids at doses of 200 and 400 × 10^−7^ mol. Table D: The reduction (%) of germination and measured early growth parameters in *Zea mays* L. seedlings by ferulic and vanillic acids at doses of 200 and 400 × 10^−7^ mol. Table E: ANOVA for the reduction (%) of germination and measured early growth parameters in *Zea mays* L. by vanillic acid at doses of 200, 400 and 600 × 10^−7^ mol. Table F: The reduction (%) of germination and measured early growth parameters in *Zea mays* L. seedlings by vanillic acid applied in doses of 200, 400 and 600 × 10^−7^ mol.
(PDF)

S3 Table The results of repeated measures ANOVA for measured and calculated chlorophyll fluorescence and multispectral traits. Table A: The results of repeated measures ANOVA for measured and calculated chlorophyll fluorescence traits. Table B: The results of repeated measures ANOVA for measured and calculated multispectral traits.
(PDF)

S4 Table The results of repeated measures ANOVA with “SLICE” option for measured and calculated chlorophyll and multispectral traits. (PDF)

S5 Table The results of one-way ANOVA for the effect of ferulic acid on *Zea mays*. (PDF)

S1 File Cramer-von-Mises permutation test and germination dynamic curves of *Ambrosia artemisiifolia* L. treated with *p*-coumaric acid, vanillic acid and ferulic acid. (PDF)
