# Correction: Three inhibitory phenolic acids against common ragweed (*Ambrosia artemisiifolia* L.) had a minimal effect on maize growth *in vitro* and *in vivo*

**DOI:** 10.1371/journal.pone.0322155

**Published:** 2025-04-04

**Authors:** Laura Pismarović, Valentina Šoštarčić, Kristina Kljak, Boris Lazarević, Maja Šćepanović

There are errors in the Funding statement. The correct Funding statement is as follows: This work is supported by the Croatian Science Foundation under the project number [HRZZ-IP-2022-10-6639].

The following information is missing from the Acknowledgement section: The publication is supported by the Open Access Publication Fund of the University of Zagreb Faculty of Agriculture.
