# Supplementary material for: Three inhibitory phenolic acids against common ragweed (Ambrosia artemisiifolia L.) had a minimal effect on maize growth in vitro and in vivo
Source: PLoS One. 2024 Sep 27;19(9):e0308825. doi: 10.1371/journal.pone.0308825 (PMC11432884; doi:10.1371/journal.pone.0308825)
Supplement: S1 Table — Table A: ANOVA for the reduction (%) of measured early growth parameters and germination in Ambrosia artemisiifolia L. by phenolic acids at a dose of 200 × 10−7 mol. Table B: The reduction (%) of germination and measured early growth parameters in Ambrosia artemisiifolia L. seedlings by phenolic acids at a dose of 200 × 10−7 mol. Table C: Two-way ANOVA for the reduction (%) of measured early growth parameters and germination in Ambrosia artemisiifolia L. by vanillic and ferulic acids at doses of 200 and 400 × 10−7 mol. Table D: The reduction (%) of germination and measured early growth parameters in Ambrosia artemisiifolia L. seedlings by ferulic and vanillic acids at doses of 200 and 400 × 10−7 mol. Table E: ANOVA for the reduction (%) of germination and measured early growth parameters in Ambrosia artemisiifolia L. by vanillic acid at doses of 200, 400 and 600 × 10−7 mol. Table F: The reduction (%) of germination and measured early growth parameters in Ambrosia artemisiifolia L. seedlings by vanillic acid applied in doses of 200, 400 and 600 × 10−7 mol. (PDF) [file pone.0308825.s001.pdf]

S1

Table A. Analysis of variance for the reduction (%) of measured early growth parameters and germination in *Ambrosia artemisiifolia* L. by phenolic acids at a dose of 200 × 10^{-7} mol.

Variable		Df	SumSq	MeanSq	F value	Sig.
Radicle length	PHA	2	56606	28303.0	23.14	***
	R	2	4696	2347.9	1.92	ns
	Error	175	214024	1223.0		
Hypocotile length	PHA	2	17509.83	8754.92	12.69	***
	R	2	39794.54	19897.27	28.83	***
	Error	175	120777.9	690.16		
Fresh weight	PHA	2	420.04	210.02	0.74	ns
	R	2	1249.26	624.63	2.21	ns
	Error	13	3677.97	282.92		
Germination	PHA	2	270.05	135.02	1.20	ns
	R	2	159.69	79.85	0.71	ns
	Error	13	1457.95	112.15		

PHA = phenolic acid (vanillic acid, ferulic acid, *p*-coumaric acid), R – repetition (3), Df = degrees of freedom, SumSq = sum of squares, MeanSq = mean of squares, Sig. = significant difference in means; (ns) not significant, (*) p = 0.05, (***) p < 0.001.

Table B. The reduction (%) of germination and measured early growth parameters in *Ambrosia artemisiifolia* L. seedlings by phenolic acids at a dose of 200 × 10^{-7} mol.

Variable	Phenolic acid	EMM	SE	DF	Lower CL	Upper CL	Tukey
Radicle length	FA	27.49	4.69	59.30	18.11	36.88	a
	VA	38.59	4.63	57.11	29.31	47.87	a
	PCA	69.41	4.28	57.90	60.84	77.98	b
Hypocotile length	FA	37.19	3.85	62.70	30.23	45.60	a
	VA	42.29	3.91	59.00	34.47	50.11	a
	PCA	60.68	3.91	62.26	52.86	68.49	b
Fresh weight	FA	40.3	2.57	5.49	33.8	46.7	NA
	VA	44.0	8.92	4.71	20.6	67.3	NA
	PCA	51.9	8.84	4.64	28.6	75.1	NA
Germination	FA	7.23	3.32	5.03	-1.28	15.7	NA
	VA	15.38	4.73	5.08	3.28	27.5	NA
	PCA	7.10	4.53	5.08	-4.49	18.7	NA

FA-ferulic acid, VA – vanillic acid, PCA – *p*-coumaric acid, EMM – estimated marginal mean, SE – standard error, DF – degree of freedom, lower/upper CL – confidence interval, NA – not applicable. Means followed by the same letter within the Tukey column are not significantly different according to the Tukey test (p < 0.05).

Table C. **Two-way analysis of variance for the reduction (%) of measured early growth parameters and germination in *Ambrosia artemisiifolia* L. by vanillic and ferulic acids at doses of 200 and 400 × 10^{-7} mol.**

Variable		Df	SumSq	MeanSq	F value	Sig.
Radicle length	PHA	1	7289	7288.8	5.76	*
	D	1	2796	2796.2	2.21	ns
	R	2	1772	885.9	0.70	ns
	PHA:D	1	0	0.3	0.0003	ns
	Error	234	296324	1266.3		
Hypocotile length	PHA	1	34	33.5	0.05	ns
	D	1	9860	9859.9	14.45	***
	R	2	45703	22851.6	33.49	***
	PHA:D	1	1574	1573.9	2.31	ns
	Error	234	159689	682.4		
Fresh weight	PHA	1	327.1	327.08	1.14	ns
	D	1	570.4	570.37	1.99	ns
	R	2	817.5	408.76	1.42	ns
	PHA:D	1	105.9	105.92	0.37	ns
	Error	18	5170.7	287.26		
Germination	PHA	1	11.15	11.15	0.16	ns
	D	1	130.48	130.48	1.84	ns
	R	2	610.39	305.20	4.31	*
	PHA:D	1	13.35	13.35	0.19	ns
	Error	18	1273.65	70.76		

PHA = phenolic acid (vanillic acid, ferulic acid), D- dose (200 and 400 × $10^{-}7$ mol), R – repetition (3), Df = degrees of freedom, SumSq = sum of squares, MeanSq = mean of squares, Sig. = significant difference in means; (ns) not significant, (*) p = 0.05, (***) p < 0.001.

Table D. The reduction (%) of germination and measured early growth parameters in *Ambrosia artemisiifolia* L. seedlings by ferulic and vanillic acids at doses of 200 and 400 × 10^{-7} mol.

Variable	Phenolic acid	Dose	EMM	SE	DF	Lower CL	Upper CL	Tukey
*Radicle length	FA	NA	30.9	3.25	117	24.5	37.4	a
	VA		42.0	3.24	118	35.6	48.4	b
Hypocotile length	NA	200	40.1	2.52	117	35.1	45.1	b
		400	27.3	2.26	116	22.8	31.8	a
Fresh weight	FA	200	41.4	4.59	10.8	31.3	51.5	NA
		400	33.6	4.59	10.8	23.5	43.7	NA
	VA	200	48.8	6.53	13.5	34.7	62.8	NA
		400	41.0	6.53	13.5	27.0	55.0	NA
Germination	FA	200	6.58	2.77	11.4	0.52	12.6	NA
		400	11.05	2.77	11.4	4.98	17.1	NA
	VA	200	7.95	3.03	12.2	1.36	14.5	NA
		400	12.41	3.03	12.2	5.82	19.0	NA

FA-ferulic acid, VA – vanillic acid, EMM – estimated marginal mean, SE – standard error, DF – degree of freedom, lower/upper CL – confidence interval, NA – not applicable. The EMMs for reduction (%) of

radicle length were averaged over phenolic acids (FA and VA) and for the reduction of hypocotyl length over applied doses (200 and 400 × 10^{-7} mol). Means followed by the same letter within the Tukey column are not significantly different according to the Tukey test ($p < 0.05$). *Results are averaged over the levels of dose and repetition.

Table E. Analysis of variance for the reduction (%) of germination and measured early growth parameters in *Ambrosia artemisiifolia* L. by vanillic acid at doses of 200, 400 and 600 × 10^{-7} mol.

Variable		Df	SumSq	MeanSq	F value	Sig.
	D	2	10248	5124.0	4.19	*
Radicle length	R	2	1122	560.8	0.46	ns
	Error	175	214089	1223.4		
	D	2	11058	5528.9	8.11	***
Hypocotyl length	R	2	27682	13840.8	20.30	***
	Error	175	119293	681.7		
	D	2	1596.4	798.21	2.25	ns
Fresh weight	R	2	95.5	47.75	0.14	ns
	Error	13	4617.1	355.16		
	D	2	224.85	112.42	0.92	ns
Germination	R	2	220.24	110.12	0.90	ns
	Error	13	1593.73	122.59		

D - dose (200, 400 and 600 × 10^{-7} mol); Df = degrees of freedom, SumSq = sum of squares, MeanSq = mean of squares, Sig. = significant difference in means; (ns) not significant, (*) $p = 0.05$, (***) $p < 0.001$.

Table F. The reduction (%) of germination and measured early growth parameters in *Ambrosia artemisiifolia* L. seedlings by vanillic acid applied in doses of 200, 400 and 600 × 10^{-7} mol.

Variable	Dose	EMM	SE	DF	Lower CL	Upper CL	Tukey
	200	38.6	4.71	57.3	29.2	48.0	a
Radicle length	400	45.3	4.44	59.2	36.5	54.2	ab
	600	56.9	4.39	57.8	48.1	65.7	b
	200	42.3	3.26	57.4	35.8	48.8	b
Hypocotile length	400	24.3	3.31	56.8	17.7	31.0	a
	600	27.4	3.54	58.6	20.3	34.5	a
	200	58.2	3.11	3.53	49.1	67.3	NA
Fresh weight	400	45.5	9.43	4.93	21.2	69.8	NA
	600	35.2	8.19	4.91	14.0	56.4	NA
	200	7.1	4.76	2.66	-9.22	23.4	NA
Germination	400	13.3	4.66	4.29	0.70	25.9	NA
	600	15.5	4.13	2.99	2.28	28.6	NA

EMM – estimated marginal mean, SE – standard error, DF – degree of freedom, lower/upper CL – confidence interval, NA – not applicable. Means followed by the same letter within the Tukey column are not significantly different according to the Tukey test ($p < 0.05$).
