# Supplementary material for: Three inhibitory phenolic acids against common ragweed (Ambrosia artemisiifolia L.) had a minimal effect on maize growth in vitro and in vivo
Source: PLoS One. 2024 Sep 27;19(9):e0308825. doi: 10.1371/journal.pone.0308825 (PMC11432884; doi:10.1371/journal.pone.0308825)
Supplement: S2 Table — Table A: ANOVA for the reduction (%) of measured early growth parameters and germination in Zea mays L. by phenolic acids at a dose of 200 × 10−7 mol. Table B: The reduction (%) of germination and measured early growth parameters in Zea mays L. seedlings by phenolic acids at a dose of 200 × 10−7 mol. Table C: Two-way ANOVA for the reduction (%) of measured early growth parameters and germination in Zea mays L. by vanillic and ferulic acids at doses of 200 and 400 × 10−7 mol. Table D: The reduction (%) of germination and measured early growth parameters in Zea mays L. seedlings by ferulic and vanillic acids at doses of 200 and 400 × 10−7 mol. Table E: ANOVA for the reduction (%) of germination and measured early growth parameters in Zea mays L. by vanillic acid at doses of 200, 400 and 600 × 10−7 mol. Table F: The reduction (%) of germination and measured early growth parameters in Zea mays L. seedlings by vanillic acid applied in doses of 200, 400 and 600 × 10−7 mol. (PDF) [file pone.0308825.s002.pdf]

S2

Table A. Analysis of variance for the reduction (%) of measured early growth parameters and germination in *Zea mays* L. by phenolic acids at a dose of 200 × 10^{-7} mol.

Variable		Df	SumSq	MeanSq	F value	Sig.
Radicle length	PHA	2	1442	721.0	1.96	ns
	R	2	11061	5530.6	15.01	***
	Error	175	64473	368.4		
Coleoptile length	PHA	2	1552.7	761.37	5.1629	**
	R	2	1248.9	624.44	4.2343	*
	Error	175	25807.4	147.47		
Fresh weight	PHA	2	124.19	62.10	1.45	ns
	R	2	59.44	29.72	0.70	ns
	Error	13	555.17	42.71		
Germination	PHA	2	270.05	135.03	1.20	ns
	R	2	159.69	79.85	0.71	ns
	Error	13	1457.95	112.15		

PHA = phenolic acid (vanillic acid, ferulic acid, *p*-coumaric acid), R – repetition (3), Df = degrees of freedom, SumSq = sum of squares, MeanSq = mean of squares, Sig. = significant difference in means; (ns) not significant, (*) p = 0.05, (**) p = 0.01, (***) p < 0.001.

Table B. The reduction (%) of germination and measured early growth parameters in *Zea mays* L. seedlings by phenolic acids at a dose of 200 × 10^{-7} mol.

Variable	Phenolic acid	EMM	SE	DF	Lower CL	Upper CL	Tukey
	FA	16.1	3.20	59.1	9.7	22.5	NA
Radicle length	VA	10.1	2.16	59.1	5.8	14.5	NA
	PCA	16.2	2.53	59.4	11.1	21.2	NA
Coleoptile length	FA	5.83	1.40	58.9	3.04	8.63	a
	VA	12.12	1.95	58.9	8.21	16.02	b
	PCA	6.07	1.37	59.00	3.32	8.81	a
	FA	3.48	2.67	13	-2.28	9.24	NA
Fresh weight	VA	6.43	2.67	13	0.66	12.19	NA
	PCA	0.00	2.67	13	-5.76	5.76	NA
	FA	7.23	3.69	4.48	-2.60	17.1	NA
Germination	VA	7.10	4.67	4.06	-5.79	-5.79	NA
	PCA	15.38	4.51	3.65	2.37	28.4	NA

FA-ferulic acid, VA – vanillic acid, PCA – *p*-coumaric acid, EMM – estimated marginal mean, SE – standard error, DF – degree of freedom, lower/upper CL – confidence interval, NA – not applicable. Means followed by the same letter within a Tukey column are not significantly different according to the Tukey test (p < 0.05).

Table C. Two-way analysis of variance for the reduction (%) of measured early growth parameters and germination for *Zea mays* L. by vanillic and ferulic acids at doses of 200 and 400 × 10^{-7} mol.

Variable		Df	SumSq	MeanSq	F value	Sig.
Radicle length	PHA	1	3997	3997.0	12.63	***
	D	1	247	246.5	0.78	ns
	R	2	14796	7398.0	23.37	***
	PHA:D	1	286	286.1	0.90	ns
	Error	234	74071	316.5		
Coleoptile length	PHA	1	1205	1204.81	8.85	**
	D	1	361	360.76	2.65	ns
	R	2	1657	828.66	6.09	**
	PHA:D	1	195	194.89	1.43	ns
	Error	234	31850	136.11		
Fresh weight	PHA	1	12.28	12.28	0.22	ns
	D	1	11.50	11.50	0.20	ns
	R	2	126.04	63.02	1.11	ns
	PHA:D	1	114.98	114.98	2.02	ns
	Error	18	1025.00	56.94		
Germination	PHA	1	24.00	24.00	1.27	ns
	D	1	0.00	0.00	0.00	ns
	R	2	37.33	18.67	0.98	ns
	PHA:D	1	10.67	10.67	0.56	ns
	Error	18	341.33	18.96		

PHA = phenolic acid (vanillic acid, ferulic acid), D- dose (200 and 400 × 10-7 mol), R – repetition (3), Df = degrees of freedom, SumSq = sum of squares, MeanSq = mean of squares, Sig. = significant difference in means; (ns) not significant, (*) p = 0.05, (***) p < 0.001.

Table D. The reduction (%) of germination and measured early growth parameters in *Zea mays* L. seedlings by ferulic and vanillic acid at doses of 200 and 400 × 10^{-7} mol.

Variable	Phenolic acid	Dose	EMM	SE	DF	Lower CL	Upper CL	Tukey
*Radicle length	FA	NA	16.18	1.78	104	12.66	19.7	b
	VA		8.04	1.46	103	5.14	10.9	a
*Coleoptile length	FA	NA	5.51	0.94	117	3.66	7.36	a
	VA		9.99	1.18	118	7.65	12.33	b
Fresh weight	FA	200	6.66	3.06	12.8	0.04	13.28	NA
		400	3.29	3.06	12.8	-3.32	9.91	NA
	VA	200	5.23	2.18	10.0	0.37	10.09	NA
		400	1.86	2.18	10.0	-3.00	6.73	NA
Germination	FA	200	2.19	1.70	12.99	-1.49	5.88	NA
		400	3.14	1.70	12.99	-0.54	6.80	NA
	VA	200	0.19	0.90	9.65	-1.811	2.20	NA
		400	1.14	0.90	9.65	-0.866	3.14	NA

FA-ferulic acid, VA – vanillic acid, EMM – estimated marginal mean, SE – standard error, DF – degree of freedom, lower/upper CL – confidence interval, NA – not applicable. The EMMs for reduction (%) of radicle length were averaged over phenolic acids (FA and VA) and for the reduction of hypocotyl length over applied doses (200 and 400 × 10^{-7} mol). Means followed by the same letter within the Tukey column are not significantly different according to the Tukey test (p < 0.05). *Results are averaged over the levels of dose and repetition.

Table E. Analysis of variance for the reduction (%) of germination and measured early growth parameters in *Zea mays* L. by vanillic acid at doses of 200, 400 and 600 × 10^{-7} mol.

Variable		Df	SumSq	MeanSq	F value	Sig.
Radicle length	D	2	889	444.71	1.70	ns
	R	2	2767	1383.43	5.27	**
	Error	175	45902	262.30		
Coleoptile length	D	2	1620.5	810.25	5.06	**
	R	2	536.0	267.99	1.67	ns
	Error	175	28022.1	160.13		
Fresh weight	D	2	87.28	43.64	1.02	ns
	R	2	31.81	15.91	0.39	ns
	Error	12	488.54	40.71		
Germination	D	2	7.11	3.56	1	ns
	R	2	7.11	3.56	1	ns
	Error	13	46.22	3.56		

D - dose (200, 400, 600 × 10^{-7} mol); Df = degrees of freedom, SumSq = sum of squares, MeanSq = mean of squares, Sig. = significant difference in means; (ns) not significant, (**) p = 0.01, (***) p < 0.001.

Table F. The reduction (%) of germination and measured early growth parameters in *Zea mays* L. seedlings by vanillic acid applied in doses of 200, 400, and 600 × 10^{-7} mol.

Variable	Dose	EMM	SE	DF	Lower CL	Upper CL	Tukey
Radicle length	200	10.13	2.17	58.7	5.79	14.47	NA
	400	5.91	1.78	58.0	2.35	9.47	NA
	600	11.00	2.29	59.0	6.42	15.58	NA
Coleoptile length	200	12.12	1.91	57.2	8.29	15.95	b
	400	7.86	1.43	54.7	5.00	10.72	ab
	600	4.80	1.52	55.0	1.75	7.85	a
Fresh weight	200	6.43	2.93	5.15	-1.05	13.90	NA
	400	0.73	0.66	2.31	-1.76	3.23	NA
	600	4.34	3.02	4.91	-3.47	12.16	NA
Germination	200	0.00	0.77	13	-1.66	1.66	NA
	400	1.33	0.77	13	-0.33	3.00	NA
	600	0.00	0.77	13	-1.66	1.66	NA

EMM – estimated marginal mean, SE – standard error, DF – degree of freedom, lower/upper CL – confidence interval. Means followed by the same letter within the Tukey column are not significantly different according to the Tukey test (p < 0.05).
