# Supplementary material for: Three inhibitory phenolic acids against common ragweed (Ambrosia artemisiifolia L.) had a minimal effect on maize growth in vitro and in vivo
Source: PLoS One. 2024 Sep 27;19(9):e0308825. doi: 10.1371/journal.pone.0308825 (PMC11432884; doi:10.1371/journal.pone.0308825)
Supplement: S3 Table — Table A: The results of repeated measures ANOVA for measured and calculated chlorophyll fluorescence traits. Table B: The results of repeated measures ANOVA for measured and calculated multispectral traits. (PDF) [file pone.0308825.s003.pdf]

S3

Table A. The results of repeated measures ANOVA for measured and calculated chlorophyll fluorescence traits.

SOV	n-1	F0	Fm	Fv/Fm	Fs'	Fm'	Fq'/Fm'	rETR	NPQ	F0'	qP	qN	qL	ϕno	ϕnpq	npq(t)
DOM	6	<.0001*	<.0001*	<.0001*	<.0001*	<.0004*	0.0015*	<.0001*	0.0003*	<.0001*	0.0174*	0.0070*	0.5941	<.0001*	0.0013*	<.0001*
T	3	0.1902	0.417	0.0732	0.3616	0.2194	0.2154	0.3571	0.1052	0.1812	0.0587	0.1015	0.0725	0.3281	0.1487	0.2029
DOM x T	18	<.0001*	<.0001*	0.2544	<.0001*	0.0049*	0.4401	0.6223	0.0024*	0.0002*	<.0001*	0.0212*	<.0001*	0.0011*	0.0882*	<.0001*

SOV - Source of variablity; DOM - day of measurement; T - treatment. P-values showing a significant difference at 0.05 significance level are denoted with asterisk (*).

Table B. The results of repeated measures ANOVA for measured and calculated multispectral traits.

SOV	n-1	Red	Green	Blue	Hue	Saturation	Value	SpcGrn	FarRed	Nir	ChlIdx	AriIdx	NDVI
DOM	6	<.0001*	0.1059	<.0001*	<.0001*	<.0001*	0.3998	0.0248*	<.0001*	<.0001*	0.0036*	0.0875	<.0001*
T	3	0.6912	0.8511	0.4558	0.6832	0.6682	0.7971	0.731	0.9596	0.1926	0.1968	0.4773	0.1359
DOM x T	18	0.0430*	0.1058	0.0008*	0.854	0.1839	0.1007	0.0010*	0.0014*	<.0001*	<.0001*	0.0001*	<.0001*

SOV - Source of variablity; DOM - day of measurement; T - treatment. P-values showing a significant difference at 0.05 significance level are denoted with asterisk (*).
