# Supplementary material for: Three inhibitory phenolic acids against common ragweed (Ambrosia artemisiifolia L.) had a minimal effect on maize growth in vitro and in vivo
Source: PLoS One. 2024 Sep 27;19(9):e0308825. doi: 10.1371/journal.pone.0308825 (PMC11432884; doi:10.1371/journal.pone.0308825)
Supplement: S4 Table — (PDF) [file pone.0308825.s004.pdf]

S4

Table. The results of repeated measures ANOVA with "SLICE" option for measured and calculated chlorophyll fluorescence and multispectral traits.

DOM	Fm	NPQ	qP	Blue	SpcGrn	Nir	NDVI
T1	0.0088*	0.1545	0.0081*	0.4925	0.9618	0.1692	0.1186
T2	0.0089*	0.2156	0.0554	0.5132	0.3403	0.2362	0.4805
T3	0.2708	0.3903	0.1625	0.4667	0.8061	0.1389	0.2587
T4	0.2175	0.4705	0.0682	0.3096	0.6667	0.1364	0.0831
T5	0.4376	0.3406	0.1987	0.1598	0.1738	0.7537	0.1011
T6	0.7722	0.1139	0.1279	0.2743	0.3186	0.1416	0.0602
T7	0.8664	0.0342*	0.0715	0.6461	0.5435	0.0888	0.0044*

DOM - day of measurement; T1-T7 - measurements were taken through seven consecutive days with T1 being the first and T7 being the last day of measurement.
P-values showing a significant difference at 0.05 significance level are denoted with asterisk (*).
