# Supplementary material for: Three inhibitory phenolic acids against common ragweed (Ambrosia artemisiifolia L.) had a minimal effect on maize growth in vitro and in vivo
Source: PLoS One. 2024 Sep 27;19(9):e0308825. doi: 10.1371/journal.pone.0308825 (PMC11432884; doi:10.1371/journal.pone.0308825)
Supplement: S5 Table — (PDF) [file pone.0308825.s005.pdf]

S5

Table. The results of one-way ANOVA for the effect of ferulic acid on *Zea mays* on calculated chlorophyll fluorescence and multispectral traits.

Fm	NPQ	qP	Blue	SpcGrn	Nir	NDVI
0.9631	0.7803	0.9794	0.6467	0.6068	0.5848	0.8783

The p-values represent the significance of the effects of ferulic acid.
