# Supplementary material for: Three inhibitory phenolic acids against common ragweed (Ambrosia artemisiifolia L.) had a minimal effect on maize growth in vitro and in vivo
Source: PLoS One. 2024 Sep 27;19(9):e0308825. doi: 10.1371/journal.pone.0308825 (PMC11432884; doi:10.1371/journal.pone.0308825)

S1 File

Germination dynamics of *Ambrosia artemisiifolia* L.

```
Permutation test based on a Cramer-von-Mises type distance (Barreiro-Ures e
t al., 2019)
NULL HYPOTHESIS: time-to-event curves are equal

    level   n         D
1 Control 200 0.5873787
2     PCA 200 2.7438876
3   FA200 200 0.4116298
4   FA400 209 1.0959926
5   VA200 200 0.6627355
6   VA400 200 0.1778419
7   VA600 191 1.1836266

Observed D value =  0.9804
P value =  1
```

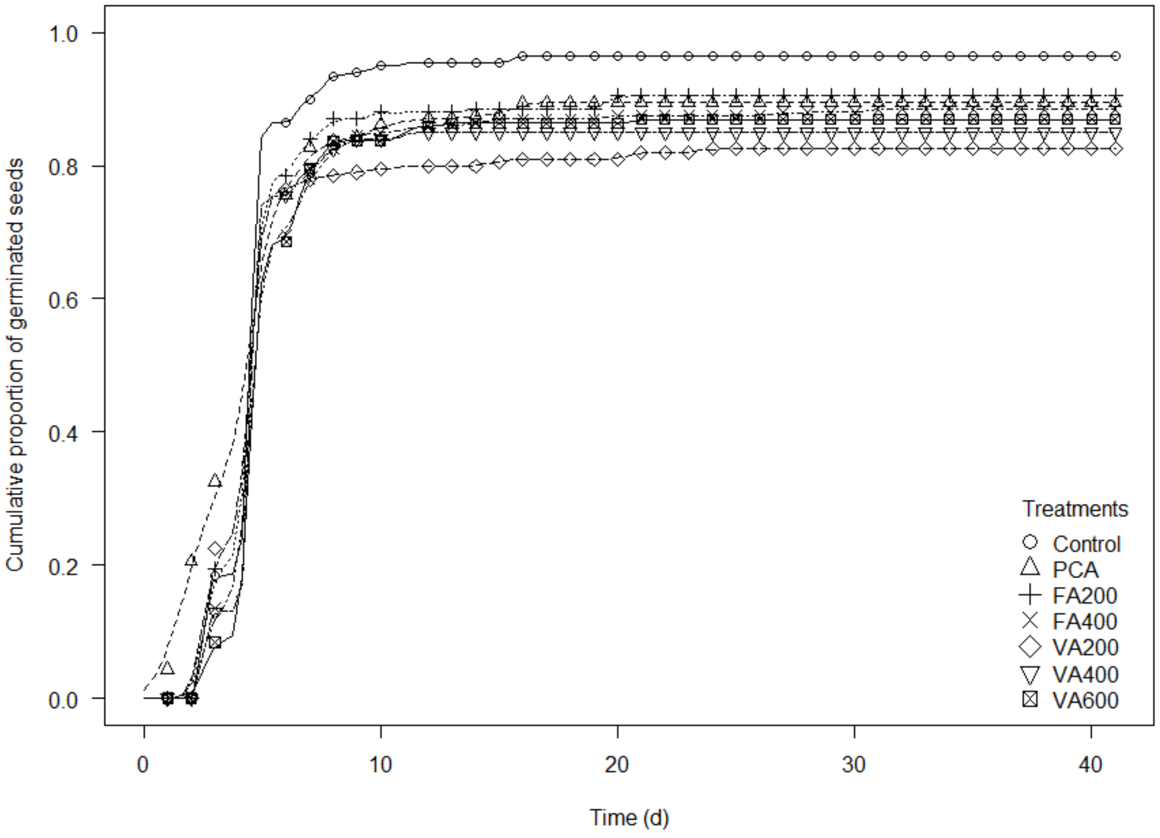

Supplement: S1 File — (PDF) [file pone.0308825.s006.pdf]
